# Cholinergic Stress Signals Accompany MicroRNA-Associated Stereotypic Behavior and Glutamatergic Neuromodulation in the Prefrontal Cortex

**DOI:** 10.3390/biom10060848

**Published:** 2020-06-03

**Authors:** Gilli Moshitzky, Shai Shoham, Nimrod Madrer, Amir Mouhammed Husain, David S. Greenberg, Raz Yirmiya, Yoram Ben-Shaul, Hermona Soreq

**Affiliations:** 1 The Institute of Life Sciences and The Edmond and Lily Safra Center of Brain Science, The Hebrew University of Jerusalem, Jerusalem 9190401, Israel; gilli.moshitzky@mail.huji.ac.il (G.M.); nm.isramail@gmail.com (N.M.); Amir.Husain@mail.huji.ac.il (A.M.H.); david.greenberg1@mail.huji.ac.il (D.S.G.); 2 Herzog Medical Center, Givat Shaul, P.O. Box 3900, Jerusalem 9103702, Israel; shaisho.shoham09@gmail.com; 3 Department of Psychology, The Hebrew University of Jerusalem, Jerusalem 9190401, Israel; raz.yirmiya@mail.huji.ac.il; 4 Department of Medical Neurobiology, The Institute of Medical Research Israel-Canada, Jerusalem 9112102, Israel; yoramb@ekmd.huji.ac.il

**Keywords:** acetylcholinesterase, cholinergic, glutamatergic, hippocampus, prefrontal cortex, microRNAs, motor control, stereotypic behavior

## Abstract

Stereotypic behavior (SB) is common in emotional stress-involved psychiatric disorders and is often attributed to glutamatergic impairments, but the underlying molecular mechanisms are unknown. Given the neuro-modulatory role of acetylcholine, we sought behavioral-transcriptomic links in SB using TgR transgenic mice with impaired cholinergic transmission due to over-expression of the stress-inducible soluble ‘readthrough’ acetylcholinesterase-R splice variant AChE-R. TgR mice showed impaired organization of behavior, performance errors in a serial maze test, escape-like locomotion, intensified reaction to pilocarpine and reduced rearing in unfamiliar situations. Small-RNA sequencing revealed 36 differentially expressed (DE) microRNAs in TgR mice hippocampi, 8 of which target more than 5 cholinergic transcripts. Moreover, compared to FVB/N mice, TgR prefrontal cortices displayed individually variable changes in over 400 DE mRNA transcripts, primarily acetylcholine and glutamate-related. Furthermore, TgR brains presented c-fos over-expression in motor behavior-regulating brain regions and immune-labeled AChE-R excess in the basal ganglia, limbic brain nuclei and the brain stem, indicating a link with the observed behavioral phenotypes. Our findings demonstrate association of stress-induced SB to previously unknown microRNA-mediated perturbations of cholinergic/glutamatergic networks and underscore new therapeutic strategies for correcting stereotypic behaviors.

## 1. Introduction

Stereotypic behavior (SB) involves patterned, repetitive, apparently purposeless movements [1]. SB occurs in patients with Tourette’s syndrome and obsessive-compulsive disorder [2], schizophrenia [3], autism [4,5], oculo-cerebro-renal disorder of Lowe (OCRL) [6], fragile X syndrome [7], Rett’s syndrome [8] and the stereotypic movement disorder in children [9]. The underlying mechanism(s) are largely unknown, but impaired emotional stress modulation is likely involved, particularly in Tourette’s syndrome [10,11]. Tourette’s patients display enhanced frequency and intensity of stereotypic motor patterns (tics) under threatening situations [12,13], and children show increased SB in unfamiliar settings [1]. The variability among human Tourette patients precludes a thorough exploration of the underlying mechanism(s), underlining the need for animal models of this syndrome.

Approaches employing pharmacological induction suggest a major role for glutamatergic neurotransmission in SB [14,15,16], but they also affect the motor and stress-induced variables. In caged animals, both stimulus-poor environments and cage mate aggression have been implicated as causes for neurodevelopmental changes that lead to behavioral stereotypies [17]. A related hypothesis views SB as a coping behavior elicited to relieve captivity stress [18]. However, this hypothesis is inconsistent with the findings that neither chronic administration of corticotrophin releasing factor (CRF) nor transgenic overexpression of glucocorticoid receptors [19] induce SB, and why avoiding SB failed to modify hormone levels in the hypothalamic-pituitary axis [20]. Others proposed that SB reflects disorganized function in executive brain regions, and correlated cage-observed stereotypic behavior patterns (frequently exhibited by captive animals) with impaired maze performance [17]. Compatible with this hypothesis, Tourette’s and autism subjects with more severe mental retardation show higher prevalence of SB [5,21,22]. However, thus far, brains of caged animals revealed very few alterations which could indicate the molecular mechanisms underlying SB. The importance of this phenomenon, combined with the limited knowhow on its underlying processes initiated our interest in pursuing transcriptomic tools for seeking the neurotransmission processes involved in SB via a bottom-up approach.

Stress effects on higher brain functions involve neuronal activation of several neurotransmitter systems, including glutamate [23], dopamine [24] and neuromodulation of both by acetylcholine (ACh) [25,26,27,28,29,30,31,32,33,34]. However, although SB is readily induced by dopamine agonists, and is modulated by stressful experiences [35,36], neither psychiatric patients nor animals with cage stereotypies showed excessive dopaminergic neurotransmission. These findings directed our attention to the putative links between glutamate and ACh in SB, possibly via regulatory microRNAs (miRNAs).

MiRNAs are non-coding RNAs, shown to be involved in the regulation of protein expression but whose role in SB remains unclear. MiRNA stem-and-loop molecules are generated from primary transcripts that are cleaved to yield 22–25 bp mature double-stranded forms, one of which guides the complex to a partially complementary sequence often found in the 3′-untranslated region (3′-UTR) region of target genes [35,37]. The miRNA 5′-end ‘seed’ region dictates target specificity and determines whether the mRNA will be translationally repressed and if it will be cleaved. Promiscuous complementation enables one miRNA to target more than one mRNA and achieve gene-network-level regulation, and genes in specific networks share common themes that could enable targeting by a single miRNA [36]. In the central nervous system, miRNAs are involved in diverse functions including neuronal development, plasticity and cellular function. Many brain miRNAs have been found to be changed in behavioral mouse models [38,39,40], but the impact of miRNAs on SB pathogenesis is still not understood. Specifically, each miRNA may target a number of different mRNA sequences; therefore, the identification of target sequences acted on by the identified mis-regulated miRNAs has not yet been well defined. 

Identifying the neurotransmission pathways involved in SB is also challenging, as receptors for the stress-response organizing agent CRF are present on glutamatergic, dopaminergic, serotonergic, noradrenergic and cholinergic neurons, indicating complex neuro-modulatory processes. Specifically, intraventricular CRF administration increases ACh release from cholinergic terminals in several forebrain regions controlling motor behavior [26], thereby potentiating principal neuron reactions to incoming messages via nicotinic receptor activation [25]. This indicates that cholinergic signals and their modification by miRNA regulators may upregulate motor functions under stress. Supporting this notion, cholinergic neurotransmission can modulate several levels of the hierarchy of motor behavior. Notably, this often occurs in an opposing manner to glutamatergic signaling [27,31,32]. Based on this knowhow, our working hypothesis predicted that stress could promote SB by modulating the regulation of motor behavior via exchanging glutamatergic with cholinergic signaling. To test this prediction, we examined transgenic mice with enforced decline in cholinergic signaling for both SB and brain transcriptomic changes of short (seeking modified miRNAs) and long transcripts (pursuing changes in their mRNA targets). 

Our mouse model involved elevated expression levels of acetylcholinesterase (AChE), known to occur under stress [33]. This overexpression could be the effect of moderating excitatory influences on stress responses and involves alternative splicing, changing AChE properties [28,29,34]. The normally rare, soluble variant AChE-R is induced by stress via alternative splicing of AChE pre-mRNA and is a preferred target of the stress-induced miRNA-132 [30] and miRNA-125b [41]. Therefore, we hypothesized that long-term AChE-R over-expression could elicit stress-related stereotypic motor behavior, by acting on neural mechanisms leading to SB. We further predicted that these could encompass various brain regions which control defensive motor responses, including the basal ganglia, limbic brain nuclei [42,43,44,45] and brainstem regions [46]; and that this might involve marked changes in miRNAs targeted at cholinergic genes. 

We set out to test the association of modifications in cholinergic-targeting miRNAs in SB by focusing on transgenic TgR mice overexpressing the human (h)AChE-R constitutively, under the minimal promoter-enhancer of cytomegalovirus (CMV). At the behavioral level, we tested TgR mice for spontaneous SB and reaction to the muscarinic stimulator pilocarpine. At the molecular level, we performed short RNA-sequencing and mRNA transcriptomic analysis of brain tissues from TgR mice, seeking modified cholinergic transcripts and their targeting miRNAs. CMV-directed gene expression depends on neuronal activity and blocks the neuro-inflammatory NFkB response regulated by cholinergic signaling [47]. Therefore, we further examined the neuronal distribution and intensity of hAChE-R overexpression and assessed neuronal activity levels by labeling the immediate early gene c-fos, shown to associate both with SB-related neural activation [16] and with AChE-R over-production [28].

## 2. Materials and Methods

*Animals:* Control, FVB/N and TgR transgenic mice were housed in the animal colony of the Herzog hospital, with an ambient temperature of 21 ± 2 °C and with a 12:12 h light: dark cycle. Experimental procedures were approved by the ethical committee MD98.08-2 of The Hebrew University of Jerusalem.

### 2.1. Behavioural Tests

*Measurements of open field motor activity:* Spontaneous stereotypic behavior in an open field was followed from weaning (3–4 weeks of age) to adulthood (2–12 months of age). Following 30 min habituation in a holding cage, mice were placed for 5 min at the mildly stressful, brightly lit, center of a 40 × 40 cm open field with 20 cm high walls. Videotaped locomotor activity was first quantified as total time spent in locomotion. Rearing episodes were quantified to evaluate the extent of disruption of normal exploration under SB. Episodes in which the mouse-initiated locomotion (“locomotion events”) were counted. An index of asymmetry was calculated as [(left turns − half the total events)/total events] * 100. Thus, perfect symmetry would yield a value of 0 and total asymmetry—50%. For measurement of circadian motor activity, see Supplementary Material, Supplementary Figure S1.

*Neurological tests:* Mice were examined at the peak of their motor activity during the dark phase. Deficits in vestibular function were tested by holding mice by the tail, lifting and then lowering them over a metal cage top. Then, while the mouse was still, tactile stimuli to the right or left shoulder and trunk were applied using a cotton swab, and orienting to the stimulus was noted. Efficient and symmetric head and limb placement, and symmetric head orienting to tactile stimuli were taken as evidence of normal vestibular functioning.

*Assessment of context-dependent SB:* Mice were observed in a brightly lit open field on test day 1, 20 min after saline injection, returned to a holding cage for 20 min and re-observed in a dimly lit room while in their home-cage. On test day 2, mice were observed in the same sequence described above, 20 min after injection. 

*Forced swimming:* Mice were placed in a circular water container, 20 × 20 cm in diameter and depth. Following a 4 min swim, at 25 °C, mice were taken out of the water and dried under a warm lamp. Total time spent swimming, number of swim episodes adjacent to the wall, number of swims across the container; number of circles and % asymmetry in the direction of swimming calculated by the index of asymmetry (see above) were quantified from video records.

*Two unit serial maze:* Modular units placed in a series constitute this maze (85). To complete one “run”, a 22 h water-deprived mouse must choose between turning right or left to receive, at the end of the maze, a reward in the form of 40 μL of 5% sucrose and one “run” is considered complete. Then the mouse must shuttle back to the other end of the maze where it gets the same reward. There were 5 runs per session; one session per day. Quantitative measures of performance include numbers of left/right choice errors, “retrace errors”—episodes in which a mouse moves in the wrong direction (toward the end without a reward), time to complete a session (5 rewarded runs) and number of errorless runs per session (5 errorless runs is the highest performance level). 

### 2.2. Histology and Immunolabeling

Mice were euthanized 80–90 min after behavioral experiments by an intraperitoneal injection of 200 mg/kg sodium pentobarbital (“Pental”). Brains were fixed by trans-cardial perfusion with ice-cold 4% paraformaldehyde containing 4% sucrose (pH = 7.4). For cellular tests we labeled ARP, the C-terminal peptide unique for AChE-R, using a rabbit polyclonal antibody [48]. Cholinergic neurons were identified by goat anti-choline-acetyltransferase (Chemicon, Temecula, USA). General AChE was labeled with goat anti-AChE (Santa Cruz, CA, USA, antibody N19). In co-localization studies, we used secondary antibodies: fluorescein (FITC)-labeled donkey anti-rabbit to visualize hAChE-R, donkey anti-goat for choline-acetyltransferase, and streptavidin-Cy3 to visualize general AChE. hAChE-R accumulating neurons were counted in hippocampal sub-regions CA1-2 (for brevity, hereafter referred to as CA1), CA3, and dentate gyrus (DG). In addition, hAChE-R accumulating neurons were counted in the striatum (in sampled rectangular fields measuring 420 × 680 µm) and in the red nucleus and the hypothalamus. Two to three coronal brain sections were sampled at an estimated distance of 2.8–3.3 mm posterior to bregma. Images of each hippocampal sub-region were acquired and analyzed using the AnalySIS software (SIS, Germany). In situ hybridization was essentially as described [48].

### 2.3. Measurements of Open Field Motor Activity

**Figure 1:**
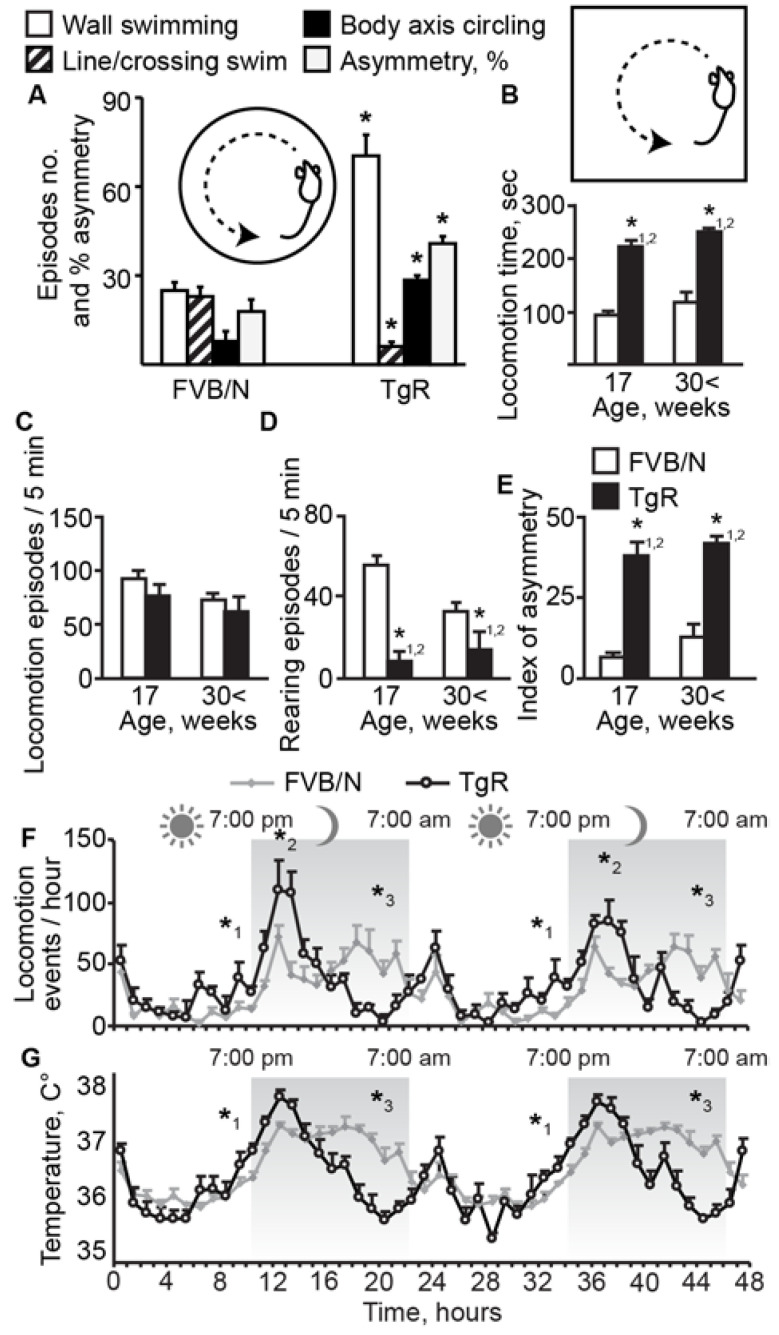
TgR mice show SB profiles. (**A**) Forced swim test. Shown are wall swim (number of swims around the circumference of the circular pool), line/crossing swim (number of episodes in which the mouse made a straight-line path instead of adhering to the wall), body axis circling (lateral circling in the water around the body axis) and asymmetry % (index of asymmetry in direction of swimming; 0 = perfect symmetry, 50% = complete asymmetry). Sample sizes: FVB/N (*n* = 10), TgR (*n* = 14). (**B**–**E**) Open field tests. Shown are locomotion time, episodes, rearing events and the index of asymmetry. (**F**,**G**) Circadian light: dark rhythm and body temperatures in TgR and FVB/N mice. Sample sizes: FVB/N (*n* = 8), TgR (*n* = 6). Asterisks represent significant ANOVA: * 1, * 2, * 3, in the first, second, and third quarters respectively.

At the age of 5 weeks, all TgR mice displayed motor activity comparable to parent strain FVB/N mice, yet by 17 weeks, they segregated into two subpopulations. About 40% of transgenics (designated TgR-L) display locomotor activity comparable to that of FVB/N controls, whereas 60% express locomotor hyperactivity (TgR-H; Table 1). The hyperactivity of TgR-H mice was manifested as locomotor asymmetry which frequently developed into stereotypic circling (Figure 1A,B), and repeating locomotor paths whereas both FVB/N and TgR-L mice readily explored and engaged in a variable succession of movements. Oral stereotypy (e.g., chewing, biting or licking) was not evident in TgR-H mice. Rearing, grooming, and the stretch-attend posture, all of which are normal components of exploratory behavior, were rare. Unlike TgR-H mice, TgR-L mice were similar to FVB/N control mice in all features (Table 1A and Supplementary Figure S1A–D). When testing behavior, we referred to the individual differences in the behavioral patterns of individual mice as significant and relevant, and separated mice into TgR-L and TgR-H as detailed below.

When the mice were single-housed in cages containing a running wheel, TgR-H mice engaged in SB at the expense of wheel running and ran the wheel considerably less than did FVB/N control mice (daily means of 2455 ± 1350 as compared to 7370 ± 1570 counts, *t*-test (df = 10) = 2.4, *p* < 0.04). Thus, SB is not due only to an increased motor drive, which could be vented by use of the running wheel. Nevertheless, the circadian locomotion cycle was essentially normal except that TgR-H mice displayed higher activity during the first half of the dark phase (Supplementary Figure S1E,F). 

Spontaneous stereotypic behavior (SB) in an open field was sought from weaning (3–4 weeks of age) to adulthood (2–12 months of age). Following 30 min of habituation in a holding cage, mice were placed for 5 min at the mildly stressful, brightly lit, center of a 40 × 40 cm open field with 20 cm high walls. Videotaped locomotor activity was first quantified as total time spent in locomotion. Rearing episodes were quantified to evaluate the extent of disruption of normal exploration under SB. Episodes in which the mouse has initiated locomotion (“locomotion events”) were counted. An index of asymmetry was calculated as: left turns—half the total events/total events *100. Thus, perfect symmetry would yield a value of 0 and total asymmetry—50%. 

### 2.4. Telemetric Measurement of Motor Activity

Telemetric activity measurements were performed as detailed previously [49], following intraperitoneal (ip) injection of ketamine–xylazine mixture (4.25, 0.15 mg/mouse) as anesthesia for transmitters implantation. Recordings lasted for 48 consecutive h starting at 7 am (beginning of light phase).

### 2.5. Vestibular Test

Mice were examined at the peak of their motor activity during the dark phase. Deficits in vestibular function were tested by holding mice by the tail, lifting and then lowering them over a metal cage top. Then, while the mouse was still, a tactile stimulus to the right or left shoulder and trunk was applied using a cotton swab, and orienting to the stimulus was noted. Efficient and symmetric head and limb placement, and symmetric head orienting to tactile stimuli were considered as evidence for normal vestibular functioning. 

### 2.6. Forced Swimming

The TgR-H phenotype extended to situations involving motor behaviors other than locomotion. Thus, in a forced swim test, TgR-H, but not TgR-L, mice swam along the wall significantly more than FVB/N mice, which tended to sample alternative routes (e.g., swimming across the container). TgR-H mice further displayed a rigid pattern of swimming direction and circling asymmetry, to the same direction as in the open field (Figure 1B). Mice were placed in a circular water container, 20 × 20 cm in diameter and depth. Following a 4 min swim at 25 °C, mice were taken out of the water and dried under a warm lamp. Total time spent swimming, number of swim episodes adjacent to the wall, number of swims across the container, number of circles and % asymmetry in the direction of swimming (calculated by the index of asymmetry) were quantified from video records. SB in this test was reflected by motor hyperactivity, reduced exploratory behavior and motor asymmetry.

### 2.7. Small RNA-Sequencing

In principle, we expected global changes in gene expression to primarily depend on the genomic composition of the tested mice. Therefore, we have used brain tissues from all of the TgR transgenics as compared to the control (wild type) mice from the same strain for the molecular analyses (microarray tests, RT-qPCR measurements and analyses thereof). Briefly, total RNA was extracted from murine brain tissues using the miRNeasy Mini Kit (Qiagen, 217004, Venlo, The Netherlands). Four TgR and three FVB/N mice were used for the prefrontal cortex (PFC) analyses, and three TgR and three FVB/N mice were used for the hippocampal preparations. RNA quality and quantity were measured using a Nanodrop 2000 (PeqLab/Thermo Scientific, Waltham, MA, USA). RNA integrity was tested using the 2100 Bioanalyzer (Agilent Technologies, Santa Clara, CA, USA) RNA 6000 Nano LabChip Kits (Agilent Technologies) which revealed a RIN values range of 8.6–9.3. 300ng of RNA were subjected to small-RNA sequencing. Libraries were generated using an RNA Library prep kit (NEBNext E7300 Multiplex Small RNA Library Prep Set for Illumina, Foster City, CA, USA) following manufacturer’s instructions. Libraries were barcoded and sequenced on a NextSeq Series Sequencing System (HUJI Center for Genomic Technologies, Jerusalem, Israel) using an Illumina flow cell (Illumina 500 NextSeq High Output v2 Kit, FC-404-2005; Illumina). Quality control was performed using FastQC [50], version 0.11.8. Short RNA was aligned to the miRBase version 21 using miRExpress2.0 [51] with default parameters. Expression analysis was performed using the Bioconductor DESeq2 [52] software via R platform [53].

### 2.8. Transcriptomic Analysis

Prefrontal cortices (PFCs) and Striatal Caudate-Putamen regions (CP) from groups of four FVB/N (wt) and four TgR animals were dissected on ice. Total PFC and CP RNA was extracted using the RNeasy mini kit (Qiagen, Hulden, Germany). Transcriptome profiles of two independent pools of PFC and CP RNAs from male age and strain-matched FVB/N and TgR mice (3–4 animals in each pool) were each hybridized to four standard Affymetrix mouse arrays (MgU74Av2) with 12,450 different transcripts for a total of 4 microarrays. Microarrays were used as per manufacturer’s instructions and the MIAME (www.mged.org) instructions. Analysis involved fluidic station FS-400, MAS 5.0 software and GeneArray 2500 scanner (Affymetrix). Transcripts were designated as absent or present according to the Affymterix manual where marginal (M) detection levels were counted as absent. A transcript was designated as changed if there was a robust change according to the Affymetrix manual (in the same direction) for at least 3 out of 4 comparisons (2 × 2 pools from each treatment). Data mining and graphs were carried out using the Spotfire program (Spotfire, Sommerville, MA, USA) and Excel. To identify the over-represented categories among those that were changed in the TgR brain, we performed Gene Ontologies (GO) classification using the freely available online GOstat tool [54]. 

### 2.9. Quantitative PCR (qPCR)

cDNA was prepared using the Quanta qScript mRNA cDNA Synthesis Kit (Quantabio, Beverly, MA, USA) according to the manufacturer’s instructions and diluted 1:10 in double-distilled water prior to qPCR plate preparation. qPCR was performed in either 384-well or 96-well plates on CFX-384/96 machines (Bio-Rad), using Perfecta Sybr Green FastMix with low or No Rox (Quantabio) at a final well volume of 5 or 15 μL, respectively. β-actin and RPL-19 were used as housekeeping genes. Expression was calculated as ΔΔCt values using the Bio-Rad CFX Maestro 1.1 Software Version 4.1.2433.1219. 

### 2.10. Statistical Analyses

Histological data was subjected to ANOVA with a simple between-groups design comparing wild type FVB/N and TgR mice. Behavioral data, which included multiple tests conducted on each mouse, was analyzed using one-way ANOVA with repeated measures within each group (FVB/N and TgR) and between groups (FVB/N vs. TgR). Other statistical tests are noted where relevant. 

## 3. Results

### 3.1. TgR Mice Show Clear SB Characteristics

At the age of 17 and 30 weeks, TgR mice displayed exacerbated motor activity compared to age-matched FVB/N controls. Although there were relatively high individual behavioral differences among TgR as compared to FVB/N mice (Table 2 and Table 3 and Supplementary Figure S1), most TgR mice exhibited symptoms that are indicative of SB, including motor hyperactivity, which was evident both in the forced swim (Figure 1A and Supplementary Movie S1) and the open-field (Figure 1B) paradigms, and was also observed under continuous automated telemetric recording. Moreover, TgR mice presented stereotypic and rigid behavioral patterns which accompanied their less than normal exploratory behaviors as observed in matched controls. For example, TgR mice exhibited stereotypic circling and swimming along the wall under forced swim stress, unlike FVB/N mice which tended to sample alternative routes (e.g., swimming across the container) (Figure 1A). In the open-field test, TgR mice presented locomotor hyperactivity at the expense of normal exploratory behaviors, reflected by marked decline in rearing. They also showed less grooming and stretch-attend postures, which are normal components of exploratory behavior (Figure 1B–D). 

Interestingly, when TgR mice were single-housed in cages containing a running wheel, they engaged in SB and ran the wheel considerably less than the FVB/N control mice (daily means of 2455 ± 1350 as compared to 7370 ± 1570 counts, *t*-test (df = 10) = 2.4, *p* < 0.04). Thus, the abnormal locomotion patterns were unlikely to be due to an increased motor drive, which could be vented by use of the running wheel. Additionally, TgR mice exhibited motor asymmetry, both in the forced swim (Figure 1A) and the open-field (Figure 1E) tests, where they displayed circling or locomotion asymmetry, turning to the same direction repeatedly. Additionally, in spite of essentially normal circadian locomotion cycle in both strains, activity levels and body temperature were both significantly higher in 2-month-old TgR mice during the first half of the dark phase (Figure 1F,G). The contribution of vestibular dysfunction and middle ear pathology to TgR circling behavior was excluded, as TgR mice displayed symmetric head orienting when touched with a probe at the shoulder and turned symmetrically toward an approaching edge. Oral stereotypy (e.g., chewing, biting or licking) was not evident in the TgR mice (Supplementary Movie S2). We conclude that TgR mice displayed persistent SB characteristics, evident as circadian controlled motion hyperactivity, excessive circling behavior and motor asymmetry patterns which were subject to impaired control of their circadian behavior patterns.

### 3.2. Muscarinic Stimulation Suppresses the Motor Hyperactivity but not the Locomotor Asymmetry Component of SB

**Figure 2:**
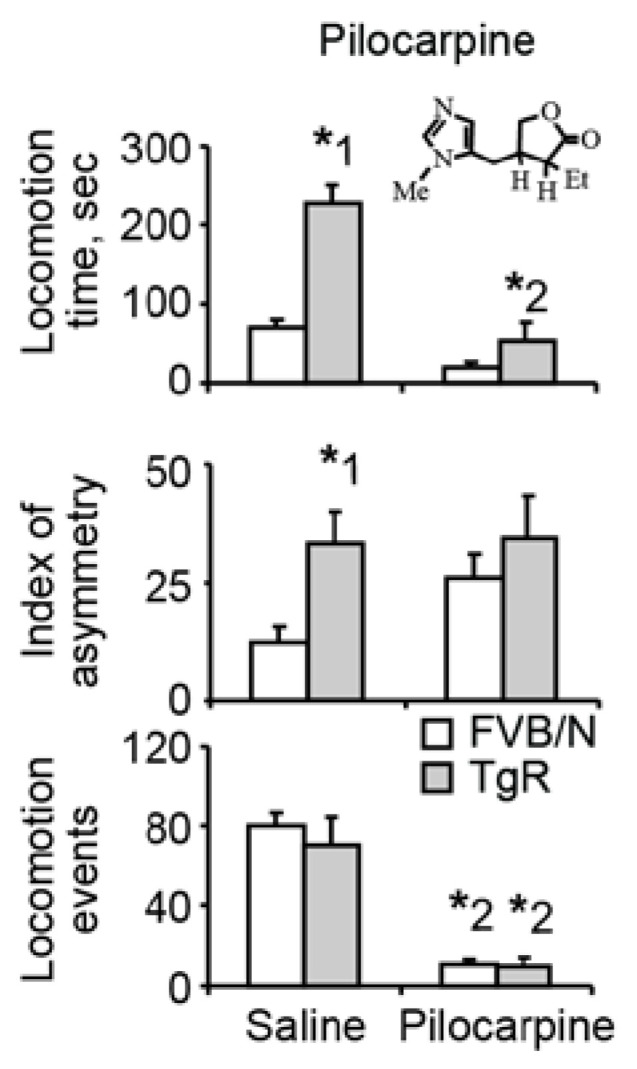
Pilocarpine suppresses the motor hyperactivity but not the locomotor asymmetry component of SB. Shown are the effects on motor activity in the open field. Pilocarpine, a muscarinic agonist, at a dose of 25 mg/kg, suppressed motor activity without affecting motor asymmetry. * 1 marks a significant transgene effect. * 2 marks a significant drug effect.

To test if the SB patterns were due to the cholinergic impairments of TgR mice, we injected these mice with the cholinergic agonist pilocarpine. This agent, at an intravenously injected dose of 25 mg/kg, profoundly suppressed motor behavior in both FVB/N and TgR mice (Figure 2 and Supplementary Table S1). Due to the higher baseline locomotion time in TgR mice, this yielded a significant interaction of pilocarpine and transgene. However, pilocarpine showed no effect on the locomotor asymmetry component of the SB profile, with no interaction of drug and transgene in this context (Supplementary Table S1), predicting that additional neurotransmission pathway(s) might be involved. Taken together, the prevalent behavioral differences between TgR and FVB/N mice, and the fact that these are manifested in multiple test conditions, suggested that these deficits did not reflect a low-level motor impairment. Rather, these findings could better be explained by a higher systematic deficit in neurotransmission pathway(s), which is what we tested next.

### 3.3. hAChE-R Excess Does Not Disrupt Normal Cholinergic and Glutamatergic Pathways and Is Inversely Correlated with ChAT Expression

**Figure 3:**
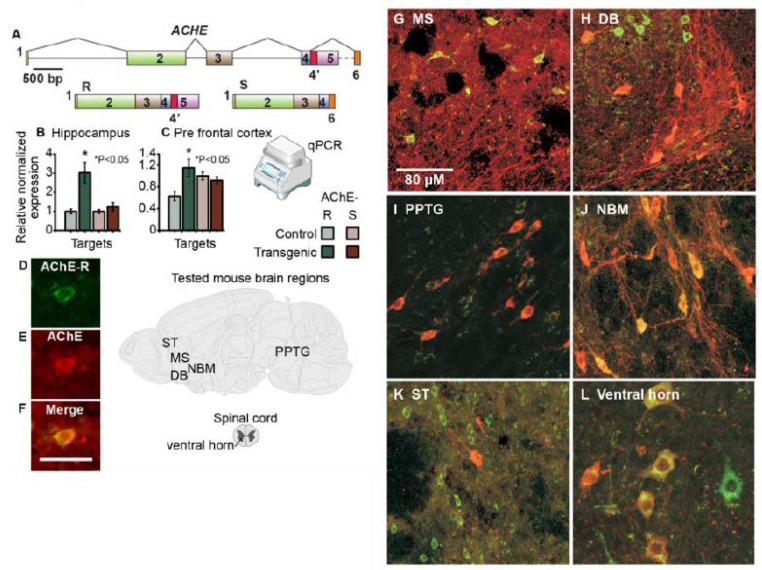
(**A**) Simplified structure of the human ACHE gene with its synaptic (AChE-S) and “readthrough” (AChE-R) mRNA 3′ alternative splicing products. (**B**,**C**). Quantitative PCR shows selective increases of the stress-induced AChE-R variant in the cholinoceptive hippocampi of TgR mice, and more modest increases of both AChE-S and AChE-R in the cholinergic prefrontal cortex. Results were normalized to RPL19. (**D**–**F**). AChE-R overexpression in matrix cells positive for the common AChE domain, but not in patches. No hAChE-R labeling appeared in cholinergic nuclei with intense choline-acetyltransferase (ChAT) staining in the medial septum (MS) (**G**), diagonal band (DB) (**H**), and the prepontine tegmental nucleus (PPTG) (**I**). hAChE-R labeling co-appeared in cholinergic nuclei expressing ChAT, with moderate to weak staining, e.g., in the nucleus basalis magnocellularis (NBM) (**J**), striatum (ST) (**K**) and spinal cord (ventral horn) (**L**). Red = ChAT, Green = hAChE-R, Yellow–Orange = co-localization of ChAT and AChE-R.

Given the cholinoceptive nature of the hippocampus and its key role in controlling navigation behavior [55,56], we have first addressed this brain region in our tests. Quantitative PCR demonstrated massive selective increases of the stress-induced AChE-R variant in the hippocampi of 10 weeks old TgR mice compared to controls, with more modest increases of both the synaptic AChE-S variant and of AChE-R in the prefrontal cortex (PFC, Figure 3A–C). Extending this analysis to the protein level, cholinergic neurons in TgR mice appeared normal in number and size and were successfully labeled for both the leading AChE-S protein and the stress-induced AChE-R variant (Figure 3D–F). Notably, TgR mice showed normal distribution of cholinergic ChAT-expressing neurons with symmetric distribution between the right and left hemispheres. However, numerous cholinergic neurons with intense ChAT expression did not express hAChE-R in the striatum, medial septum and diagonal band, indicating differential translation levels of this transcript in cholinergic neurons (Figure 3G,H,K). This also ruled out loss or impaired function of cholinergic neurons and hemispheric differences as potential causes for SB. Importantly, intensely hAChE-R-labeled neurons were denser in the medial striatum (128 ± 19 per sampled field of 100 µm^2^) than in the lateral striatum (25 ± 7 per sampled field, paired *t*-test (df = 7) = 6.8, *p* < 0.0005), opposite to the gradient of cholinergic activity in the striatum [57]. The nucleus basalis magnocellularis, brainstem and spinal cord (ventral horn) motor nuclei all included subsets of cholinergic neurons with less intense ChAT but pronounced hAChE-R expression (Figure 3I,J,L). TgR mice further showed inter-hemispheric symmetry and normal distribution of tyrosine hydroxylase-positive dopaminergic neurons, without AChE-R. Thus, the brains of TgR mice presented somewhat skewed cholinergic profiles, but with otherwise regular histopathology.

### 3.4. AChE-R Distribution in Motor-Regulating Brain Regions

**Figure 4:**
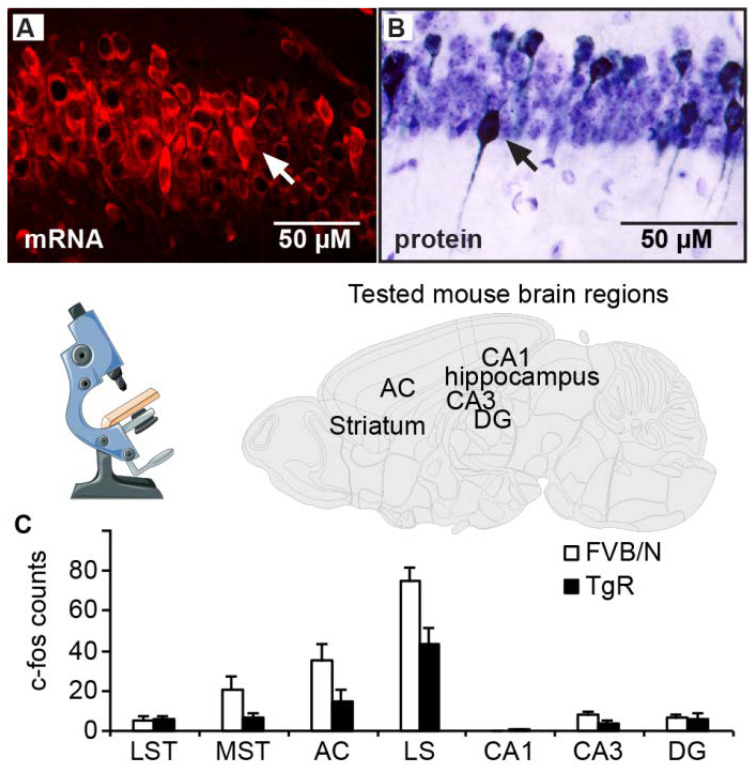
AChE-R shows hierarchical distribution in brain regions controlling motor behavior. (**A**) In FVB/N mice, subsets of CA1 neurons displayed higher than background expression of mouse AChE-R mRNA. (**B**) Neuronal activation (c-fos) patterns show inversed association to AChE-R. In the CA1 region of TgR mice, similar neuronal subsets expressed higher than background levels of the human AChE-R (hAChE-R) protein. (**C**) Using similar procedures, TgR mice presented lower basal level of neuronal activation reflected as c-fos staining in the dorsolateral striatum (LST), medial striatum (MST), anterior cingulate cortex (AC), lateral septum (LS) but not in hippocampal sub-regions CA1-2, CA3, and dentate gyrus (DG).

Multiple subsets of neurons in several brain regions of TgR mice showed an excess of both transgenic hAChE-R and murine mAChE-R mRNA, accompanied by enhanced immunolabeling of the (h)AChE-R protein. Typically, the neuronal cytoplasm, nucleus, and dendrite(s), but not axons, were stained (Figure 4A,B). In general, hAChE-R accumulation overlapped with that of the host gene, occurring in cholinoceptive regions that tend to express the primary synaptic AChE-S variant (e.g., cortex, hippocampus and striatum).

All TgR mice showed extensive hAChE-R expression in the neocortex, striatum, red nucleus and cerebellum, all of which are known to contribute to motor regulation [58,59], as well as in the anterior cingulate cortex, amygdala, hypothalamus and medial striatum, reported as being involved in the emotion-motor interface [60,61]. Some TgR mice also showed increased AChE-R expression in the striatum, lateral septum and most prominently in the hippocampus, structures known to participate in behaviorally-inhibitory pathways [62,63]. Neuronal activation was assessed by immunohistochemical detection of the immediate early gene c-fos, known to be increased under numerous insults [64,65]. Notably, the medial striatum, lateral septum and anterior cingulate cortex in TgR mice included fewer c-fos positive cells than in FVB/N mice (Figure 4C and Table 4). Thus, the observed SB patterns could not be attributed to elevated c-Fos levels.

### 3.5. RNA-Seq Reveals Modified CholinomiRs in TgR Hippocampi

**Figure 5:**
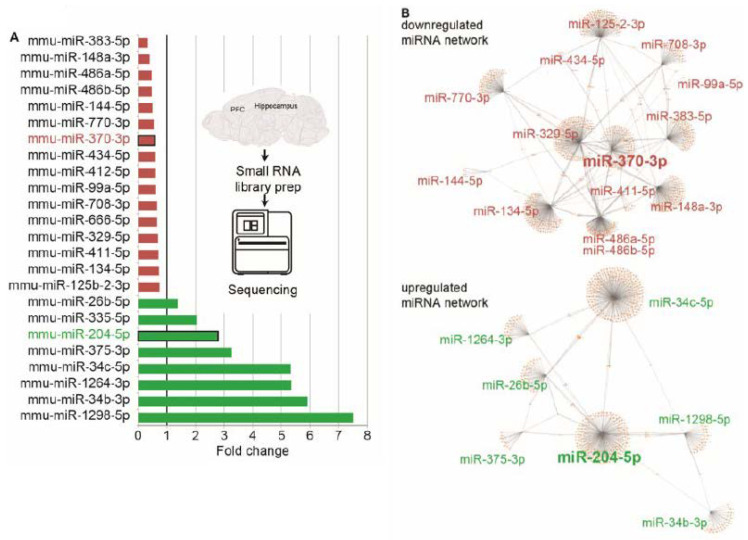
**A**. DE miRNAs in the TgR Hippocampus. Enriched DE miRNAs out of global miRNAs in the TgR hippocampus (minimal average expression of 300 normalized counts). Eight miRNAs targeting at least 5 cholinergic genes each and which are also known to be expressed in humans were DE in the TgR hippocampus, out of a total of 36 DE miRNAs. **B**. Network analysis for down- and up-regulated DE cholinergic-targeting miRNAs in the TgR hippocampus. Analysis was done using miRwalk 3.0 using maximum binding *p*-value of 1 and filter to 3’UTR binding and using miRDB database [73].

The hippocampus coordinates numerous behavioral patterns [66], and it readily reacts to altered cholinergic cues [67] and shows corresponding changes in miRNAs [68]. To explore such changes in the TgR hippocampus, we performed short RNA-sequencing of hippocampal RNAs from TgR mice and matched controls. A total of 36 miRNAs were differentially expressed (DE) in the TgR hippocampal RNA-seq datasets compared to FVB controls, and eight of these DE miRNAs target at least 5 cholinergic transcripts each (Figure 5A; dataset deposited as GEO GSE144022) [55]). Notably, the downregulated cholinergic-targeted miRNAs formed a multi-level network with other miRNAs, considerably more complex than the network formed by the upregulated miRNAs (Figure 5B). Those included miR-125b which targets, among other mRNAs both AChE-R and the vesicular ACh transporter (VAChT) [41], miR-370, the targets of which include the AKT signaling pathway [69] and BMP2 [70], and miR-204 which may inhibit the cholinergic-suppressible inflammation-associated NFkB signaling pathway [71]. The modified miRNAs further formed intricate networks, suggesting that they may each affect the other modified miRNAs (Figure 5B). Intriguingly, no miRNAs were DE in the prefrontal cortex (PFC) of TgR mice. However, given the power of such network changes to exert neuro-modulation impacts across the brain [72], we proceeded by outlining corresponding changes in mRNA transcripts of the prefrontal cortex, which communicates with the hippocampus.

### 3.6. The TgR PFC Shows More Variable Cholinergic Transcripts than the Caudate Putamen

**Figure 6:**
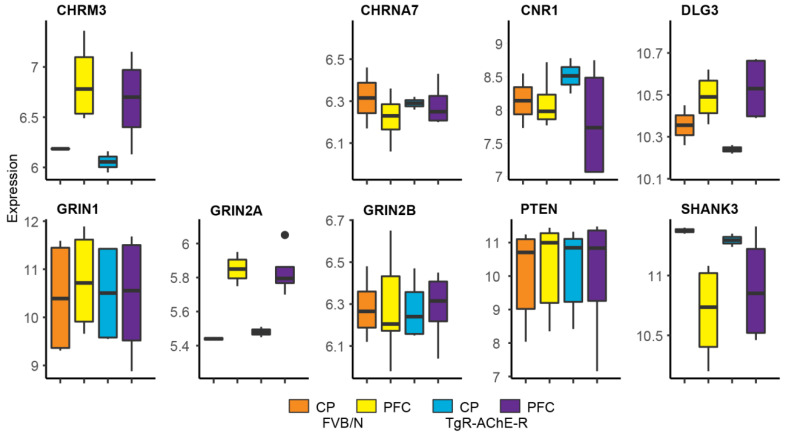
Microarray-validated individual diversity in PFC’s cholinergic-related transcripts compared to the CP. Shown are variabilities between the CP and the PFC in FVB/N mice and the TgR strain. Higher individual variability can be seen in the expression profiles between the PFC and the CP, especially in the SB-prone TgR strain.

To further explore the cholinergic signaling-related brain routes, we compared the levels of cholinergic-associated transcripts between the striatal caudate-putamen (CP) and the PFC of FVB/N control and TgR mice (dataset deposited as GEO GSE31458). Intriguingly, the PFC showed more inter-individual variability for cholinergic and stress-related transcripts including CHRM3, CHRNA7, CNR1, DLG3, GRIN2A and SHANK3 compared to their CP expression patterns (Figure 6). The greater individual diversity of cholinergic-related and stress-associated genes in the PFC compared to the CP could be functionally relevant for the SB phenotype.

### 3.7. Suppressed Glutamatergic Neurotransmission-Related Genes in the TgR PFC

**Figure 7:**
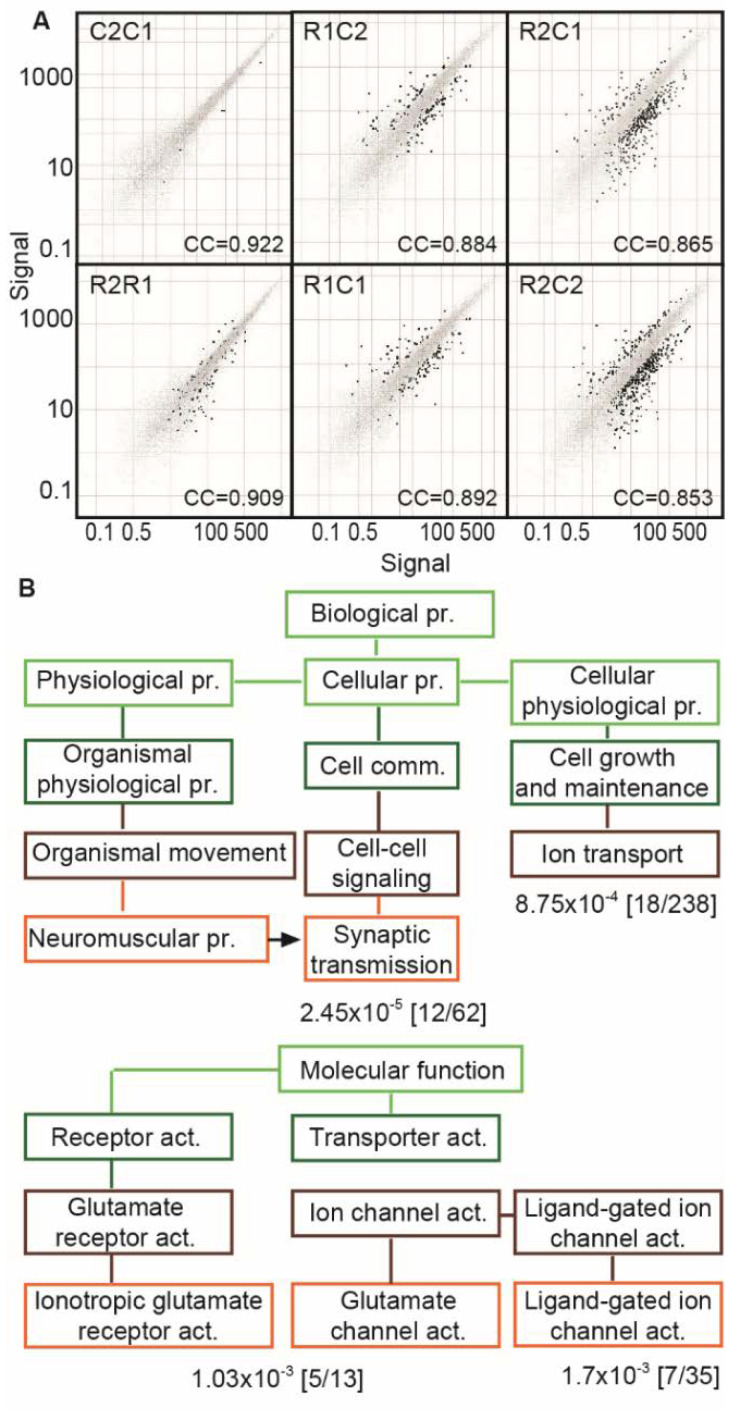
Glutamatergic transcripts are conspicuously changed in the TgR PFC. (**A**) Log10 Scatter plots demonstrating greater differences between microarray pools of 4 TgR mice PFCs (R1 R2) as compared with pools of 4 FVB/N mice PFCs (C1 C2), and yet greater differences between cross pools (R1 C2, R2 C1, R1 C1, R2 C2). Gray dots depict genes the expression level of which was unaltered between the two tests; black dots depict genes with log ratio higher than 1. (**B**) GOC pathways terms modified in TgR mice relative to parent strain mice. Terms for the biological process and molecular function are shown at the top and bottom, respectively. The lines indicate the hierarchical relationship between the terms. Values indicate the probability to observe the given number of changed transcripts within a term by chance. PFC = prefrontal cortex; GOC = gene ontology categories; pr = process; comm. = communication; act = activity.

We predicted that the miRNA changes in the TgR hippocampus reflect its cholinoceptive features; to test this hypothesis, we sought corresponding differences in miRNA-targeted coding transcripts within other brain regions that modulate the hippocampus via cholinergic signaling. Given the PFC’s capacity to send stimuli-related messages to the hippocampus [74], we performed PFC transcriptomic analyses. We compared the FVB/N vs. FVB/N (C1/C2), TgR vs. TgR (R1/R2) and the four possible combinations of FVB/N vs. TgR profiles (Figure 7A). The two FVB/N preparations yielded highly similar profiles (Figure 7B, C2 vs. C1), with merely five genes showing a change. In contrast, the two TgR microarrays displayed higher variability, with 106 transcripts designated as DE (Figure 7B, R1 vs. R2). In comparison, PFC-expressed genes in TgR mice differed from those of FVB/N mice (Figure 7B, R1 vs. C1, R2 vs. C2 or the reciprocal comparisons) by 132, 388, 143, and 428 transcripts with robust changes (also, see Figure 7B for correlation coefficients (CC) between the absence/presence calls of each of the individual arrays), especially in glutamate-related functions.

The transcriptomic tests of coding mRNAs, followed by Gene Ontology analysis (GO [54]) of the corresponding biological process and molecular function ontologies revealed several significantly downregulated cellular and organism level categories in the TgR PFC (Figure 7A). Most conspicuous were changes in transcription-related processes (e.g., DNA binding transcription factors, trans-acting transcription activators/repressors, hormone receptors and chromatin remodeling). Two major affected categories included synaptic transmission (*p* < 2 × 10^−5^, 12 of 62 genes changed) and ion transport/channel activity-related processes (*p* < 9 × 10^−4^, 18 of 238 changed). Suppressed molecular function categories included ionotropic glutamate receptor activity (1 × 10^−3^, 5/13) and glutamate-gated ion channel activity (Figure 7B). Within the category of synaptic transmission, we noted changes in CAMK2, associated with the control over long-term potentiation [75], which is indeed modified in the TgR mouse [76]; VAMP2, involved with synaptic transmission efficacy [77] and WNT7B, regulating the WNT signaling pathway [78] were also modified. The decrease in ion channel activity involved reductions in calcium, potassium, chloride and sodium channels, GABA transporter (GABRA3, [79,80]), the ATPase ATP 6V1A [81] and glutamate decarboxylase (GAD2, [82]) in addition to the glutamate receptor NR4A2 gene [83]. These are all compatible with our findings of reduced glutamate receptor levels in cultured hippocampal neurons overexpressing AChE [84,85] and reciprocal to the changes induced by excess AChE-S in PFC neurons. The changed categories are noted in Figure 7B, and the changed transcripts are listed in Supplementary Table S2.

### 3.8. Validating Brain Region-Dependent Changes in Cholinergic and SB-Related Transcripts

**Figure 8:**
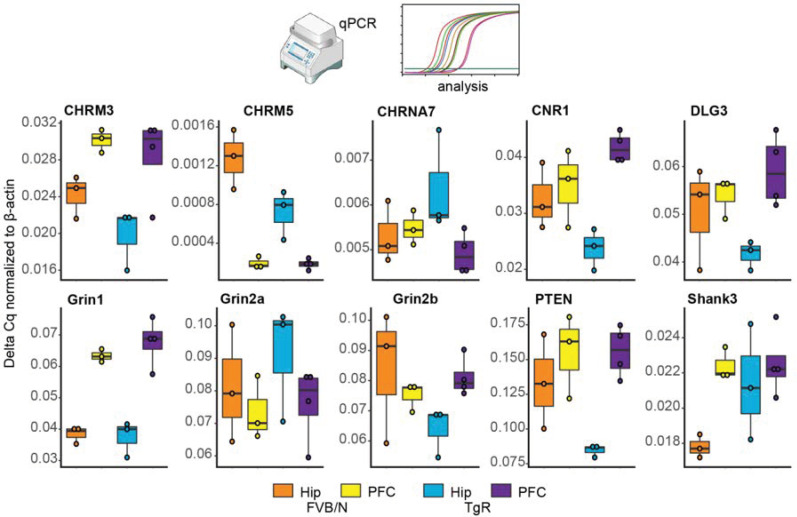
qPCR-validated change in cholinergic signaling related genes. Most changes between FVB/N mice and the TgR strain occur in the hippocampus. Different expression profiles can be observed between the PFC and hippocampus.

To validate the PFC microarray findings and seek their relevance to the over-expressed AChE-R and the modified hippocampal miRNAs, we performed qPCR tests of selected relevant mRNA transcripts which are characteristic of the predictably modified pathways in hippocampi and PFC tissues from TgR and control mice. Notably, both the muscarinic receptor CHRM3 shown to be essential for REM sleep [86] and the attention-related CHRM5 [87] showed hippocampal but not PFC differences in TgR mice compared to FVB/N controls and to unmodified transcript controls (Figure 8). Additionally, we noted massive albeit bidirectional hippocampal changes in the autism-related transcripts of Grin2b, PTEN and SHANK3 [88,89,90,91], predicting behavioral differences; but not in the inflammation-associated CHRNA7, indicating limited relevance for brain inflammation. Notably, both miR-370-3p and miR-204-5p are known controllers of brain glia, indicating their potential relevance to the observed changes in the target transcripts. We conclude that perturbation of cholinergic/glutamatergic networks may induce stress-induced miRNA-mediated SB.

## 4. Discussion

TgR mice presented locomotor SB that appeared to be a repetition of pre-potent defensive motor responses to threat. Specifically, the running component paralleled running response under threat [43,45,92], which was consistently accompanied by the TgR hyper-locomotion. This is compatible with the reported linkage between emotionality and lateralization of motor behavior [93], which may be associated in TgR mice with locomotor-asymmetry and circling. Thus, we propose that in wild type mice, escape-mode defensive pre-potent motor responses may be subjected to inhibitory control; however, in the TgR mouse, these responses are released from inhibition as revealed by c-fos reduction in executive brain regions of the TgR emotional-motor interface.

Seeking the neuronal network origin of SB and its underlying molecular mechanisms, we studied short hippocampal and long PFC RNA transcripts in SB-expressing TgR mice over-expressing the stress-inducible soluble AChE-R variant, which accelerates acetylcholine degradation and suppresses cholinergic reactions. The transcriptomic analysis was performed on a mixed group of TgR mice with predicted inter-individual variability in their SB profiles. This indicates under-estimation of the impact of this transgene on those TgR mice presenting high level SB. We found TgR hippocampi to show 36 DE miRNAs, with 8 of those targeting cholinergic-related transcripts. Moreover, the PFCs of TgR mice, which showed no DE miRNAs, displayed 428 long DE mRNA transcripts, with a conspicuous decline of glutamatergic-related pathways (*p* < 1 × 10^−3^) and pronounced changes in autism-related transcripts compared to FVB/N mice. We further noted excess of C-fos at motor behavior-regulating brain regions and of immune-labeled AChE-R in SB-regulating basal ganglia, limbic brain nuclei and the brain stem. This labeling pattern accompanied the SB profiles of TgR mice. Combined with the changes we found in hippocampal miRNAs and selected glutamatergic-related PFC transcripts, these findings may indicate miRNA-mediated perturbation of the delicate balance between cholinergic/glutamatergic networks as accompanying the impaired inhibitory control over defensive motor behavior which is causally involved in SB.

### 4.1. Modified Hippocampal miRNAs in TgR Mice with Altered Cholinergic Activities

Over-expression of the stress-inducible soluble AChE-R variant predicts excessive ACh hydrolysis and correspondingly impaired cholinergic transmission in several motor-related brain regions. This variant is over-expressed in the mammalian brain under stress responses (reviewed in [33]). Therefore, we assumed that modified expression of cholinergic miRNAs might be involved. To test this prediction, we performed non-biased profiling of the short RNAs in TgR hippocampi compared to wild type FVB/N controls. This analysis indeed revealed 36 DE miRNAs, 8 of which affect cholinergic function (‘CholinomiRs’, [94]). Those included miR-125b which targets both AChE-R and the vesicular acetylcholine transporter (VAChT) [41], indicating that transgenic over-expression of AChE-R induced miRNA-mediated suppression of both the transgene itself and the cholinergic network that it interrupts in TgR brains. Other miRNAs that were affected are miR-370, which targets the AKT signaling pathway [69] and BMP2 [70]. Changes were also observed in miR-204, which inhibits the NFkB signaling pathway that is associated with inflammation and is suppressible by cholinergic inputs [71]. Altogether, the impaired miRNA signaling in the TgR hippocampi suggests a causal involvement of the AChE-R variant in both the cholinergic pathway in general, and the SB profile of these mice in particular.

### 4.2. Prefrontal Cortex and Striatal Transcriptomics of TgR vs FVB/N Mice

The hippocampal CholinomiR changes and the neuro-modulatory role of cholinergic neurotransmission [33,95] suggested the existence of further modifications in other brain regions. Notably, the PFC showed larger variability in cholinergic-related transcripts than the striatal caudate-putamen. Given the stress-related links of PFC functioning with the hippocampus, we proceeded by transcriptomic profiling of long RNAs in the PFC. This analysis revealed greater variability in PFC transcripts from TgR mice compared to their parent FVB/N strain. Specifically, we found the categories of both signal transduction and protein kinase C activation to be modified in the TgR PFC, which indicates that AChE-R may play an active role in modulating motor responses to stress. Additionally, the neuro-anatomical distribution of AChE-R overlapped with that of its partner kinase [96], consistent with our previous studies showing intensified, PKC-mediated LTP responses in TgR mice [76,97]. Therefore, the variable neuroanatomical distribution of transgenic AChE-R may support the gene-environment interaction(s) accounting for the TgR SB phenotype. Furthermore, the altered synaptic regulation by AChE-R occurred in specific pathways involved with stress management, consistent with the overlap between AChE-R’s neuro-anatomical distribution and the distribution of stress-activated neuronal populations.

### 4.3. Implications for SB Studies

Several studies support glutamatergic causation of SB. This centers on anatomical substrates within the striatum [16,98], and possibly other brain regions in the motor hierarchy. Such conclusions are mainly derived from clinical studies. For example, Tourette’s syndrome abnormalities involve interactions between the striatum and other brain regions [99]. However, although animal model studies of spontaneous cage stereotypies [17] associated captivity stress with SB, the anatomical substrate of SB in these models has not been elucidated yet. Our current study bridges the anatomical distribution of stress signals with the corresponding transcriptomic signals and the SB phenotype, suggesting that stress-induced changes in AChE-R distribution and in neuronal activation in the behavioral-motor interface may yield SB. Importantly, SB is not a single class of purposeless movement. Rather, we find that stress-related cholinergic signaling intensifies pre-potent escape-related locomotion while other motor behaviors such as rearing are reduced. That TgR mice display SB patterns under safety supports the notion of stress-induced restriction of their normal behavioral repertoire. Different types of stress may hence engage distinct pre-potent defensive motor responses and result in different forms of SB, with glutamate involved in some but not necessarily all aspects of SB.

### 4.4. Clinical Relevance to Human SB

The elevated stress signaling [100] and reduced inhibitory tone in executive brain regions of the emotional motor interface [101,102] in Tourette’s syndrome parallel our observations in TgR mice. The frequency and intensity of tics (a stereotypic motor pattern) displayed by Tourette’s patients increase under situations perceived as threatening [12,13]. In addition, striatal cholinergic activity is involved in both human SB and psychosis. Along with the cingulate and orbito-frontal cortex, the caudate nucleus serves as the major substrate for the effects of stress on glutamatergic pathways [103]. Both the caudate and cingulate/prefrontal cortices are involved in the TgR model. Nevertheless, the movement sequences that comprise SB differ among mouse models, caged animals or psychiatric patients. While locomotor hyperactivity and circling behavior occur both in some psychiatric disorders [104] and in TgR mice, several other SB movement sequences appear in psychiatric and developmental disorders [8] but not in TgR mice. Nevertheless, the clinical relevance of cholinergic stress signaling to SB in psychiatric disorders is supported by our finding that in human blood tests, AChE-R levels increase with state anxiety [105]. We conclude that SB reflects a multitude of pre-potent movement sequences that escape inhibitory control, that are often stress-related and that vary depending on the neural substrates involved in the given psychiatric disorder. While we are aware of the limitations of this study, which largely presents an association phenomenon rather than a conclusive mechanism of action, it may be naïve to link the complex SB phenomenon to a single splice variant of a single enzyme; nevertheless, the broad distribution of cholinergic stress signaling points to AChE-R as an important candidate to investigate in psychiatric disorders with stereotypic behaviors.

## Figures and Tables

**Table 1:** Primer pairs for the tested transcripts are listed below.

Gene	Forward Primer Sequence	Reverse Primer Sequence
Shank3	CCGGACCTGCAACAAACGA	GCGCGTCTTGAAGGCTATGAT
Pten	TGGATTCGACTTAGACTTGACCT	GCGGTGTCATAATGTCTCTCAG
CHRM3	CCTCGCCTTTGTTTCCCAAC	TTGAGGAGAAATTCCCAGAGGT
CHRM5	CCTCTACACGACCTACATCCTC	GTATGTCAGTGGTCTTGTGATGG
CHRNA7	CACATTCCACACCAACGTCTT	AAAAGGGAACCAGCGTACATC
CNR1	GTGTTCCACCGCAAAGATAGT	GCCTGTGAATGGATATGTACCTG
Grin2b	CAGCAAAGCTCGTTCCCAAAA	GTCAGTCTCGTTCATGGCTAC
Grin2a	ACGTGACAGAACGCGAACTT	TCAGTGCGGTTCATCAATAACG
Grin1	AGAGCCCGACCCTAAAAAGAA	CCCTCCTCCCTCTCAATAGC
DLG3	AACAGATCGGTGTGATCCCTA	CTGTCCTGGCATGGAACTTCA
AChE-S	CTGAACCTGAAGCCCTTAGAG	CCGCCTCGTCCAGAGTAT
AChE-R	CTGAACCTGAAGCCCTTAGAG	GGGGAGGTGGAGAAGAGAG
Beta-actin	CCACACCCGCCACCAGTT	TACAGCCCGGGGAGCAT
RPL-19	GATTGACCGCCATATGTATCAC	GTCAGCCAGGAGCTTCTTG

**Table 2:** The behavioral paradigm. Behavioral tests comparing naive FVB/N, TgR-L and TgR-H mice.

Test Paradigm	Variable	F Value	Degrees of Freedom	*p* Value	Significant Post Hoc N.K. Tests, *p* < 0.05
1. Open field	Age 17 weeks				
	Locomotion time	16.5	(2.16)	0.0001	TgR-H > TgR-L, FVB/N
	Locomotion episodes			N.S.	
	Locomotor asymmetry	31.7	(2.16)	0.0001	TgR-H > TgR-L, FVB/N
	Rearing	29.6	(2.16)	0.005	TgR-H < TgR-L, FVB/N
	Age 30 weeks				
	Locomotion time	19.9	(2.32)	0.0001	TgR-H > TgR-L, FVB/N
	Locomotion episodes			N.S.	
	Locomotor asymmetry	20.7	(2.32)	0.0001	TgR-H > TgR-L, FVB/N
	Rearing	7.8	(2.32)	0.005	TgR-H < TgR-L, FVB/N
2. Forced swim	Swims along wall	18.6	(2.29)	0.0001	TgR-H > TgR-L, FVB/N
	Swims across pool	10.5	(2.29)	0.0005	TgR-H < TgR-L, FVB/N
	Body-wise circling	9.2	(2.29)	0.0005	TgR-H > TgR-L, FVB/N
	% Asymmetry	21.1	(2.29)	0.0001	TgR-H > TgR-L, FVB/N
3. Serial choice maze	Right/left choice errors	19.1	(2.47)	0.0001	TgR-H > TgR-L>FVB/N
	Retrace errors	14.0	(2.47)	0.001	TgR-H > FVB/N
	Errorless runs	11.0	(2.47)	0.0001	TgR-H < FVB/N

Comparisons were made using one-way analysis of variance (ANOVA). Entries display ANOVA test results and post-hoc Neumann–Keuls (N.K.) comparisons. Abbreviations: N.S. = main effect of transgene was not significant. The > sign denotes a value being significantly larger than the next mentioned value, the sign < denotes ‘smaller than the next value’.

**Table 3:** Circadian rhythms in naive FVB/N, TgR-L and TgR-H mice.

Biological Activity	Circadian Time Interval	F Value	Degrees of Freedom	*p* Value	Significant Post Hoc N.K. Tests, *p* < 0.05
1. Locomotion	Light phase, 1st half			N.S.	
	Light phase, 2nd half	11.2	(2.21)	0.0005	TgR-H > TgR-L, FVB/N
	Dark phase, 1st half	11.8	(2.21)	0.0005	TgR-H > TgR-L, FVB/N
	Dark phase, 2nd half	42	(2.21)	0.0001	FVB/N > TgR-H, TgR-L
2. Temperature	Light phase, 1st half	4.3	(2.21)	0.03	FVB/N > TgR-H, TgR-L
	Light phase, 2nd half	7.5	(2.21)	0.0001	FVB/N < TgR-H, TgR-L
	Dark phase, 1st half			N.S.	
	Dark phase, 2nd half	38	(2.21)	0.0005	FVB/N > TgR-H, TgR-L
3. Circadian correlation: locomotion vs. temperature	Over the entire 24 h cycle			N.S.	
	Light phase, 1st half			N.S.	
	Light phase, 2nd half			N.S.	
	Dark phase, 1st half	9.8	(2.21)	0.001	FVB/N < TgR-H, TgR-L
	Dark phase, 2nd half			N.S.	

Comparisons were made using one-way analysis of variance (ANOVA). Entries display ANOVA test results and post-hoc Neumann–Keuls (N.K.) comparisons. Abbreviations: N.S. = main effect of transgene was not significant.

**Table 4:** Neuronal c-fos activation in key structures of the emotional-motor interface.

Brain Region	F Value	*p* Value	Significant Post Hoc N.K. Tests, *p* < 0.05
Dorsolateral striatum	3.81	0.036	TgRL > TgRH, FVB/N
Medial striatum	4.47	0.0223	TgRH < TgRL, FVB/N
Anterior cingulate cortex	3.19	0.059	TgRH < FVB/N
Lateral septum	3.0	0.068	TgRH < FVB/N
Hippocampus, CA1	2.8	N.S.	
Hippocampus, CA3	2.23	N.S.	
Hippocampus, dentate gyrus	0.04	N.S.	

This table summarizes results of ANOVA comparing FVB/N (*n* = 6), TgR-L (*n* = 11) and TgR-H mice (*n* = 10). Entries are F value, F (2.24). Abbreviations: N.S. = main effect or interaction not significant.

## Supplementary Materials

The following are available online at https://www.mdpi.com/2218-273X/10/6/848/s1, Figure S1: TgR mice show modified circling behaviour. Table S1: Pharmacological experiments in the open field paradigm: cholinergic impact. Video S1: TgR mice show motor hyperactivity in forced swim. Video S2: TgR mice display symmetric head orienting.
