# Supplementary material for: Cholinergic Stress Signals Accompany MicroRNA-Associated Stereotypic Behavior and Glutamatergic Neuromodulation in the Prefrontal Cortex
Source: Biomolecules. 2020 Jun 3;10(6):848. doi: 10.3390/biom10060848 (PMC7355890; doi:10.3390/biom10060848)
Supplement: Supplementary file 1 [file biomolecules-10-00848-s001.zip › Supplementary Table 1 NEW.docx]

**Supplementary Table 1**: Pharmacological experiments in the open field paradigm: cholinergic impact.

| Drug | Variable | Transgene effect | Drug effect | Interaction of effects | Significant post hoc N.K. tests, p< 0.05 |
| --- | --- | --- | --- | --- | --- |
| 1. Pilocarpine 25 mg/kg | a. Locomotion events | N.S. | F (1,12) =68  P<0.0001 | N.S. | Pilocarpine suppressed initiation of locomotion. |
|  | b. Locomotion time | F (1,12) =32  P<0.0001 | F (1,12) =82  P<0.0001 | F (1,12) = 25  P<0.0001 | TgR > FVB/N  Pilocarpine < saline  Reduction was greater in TgR mice. |
|  | c. Locomotor asymmetry | F (1,12) =4.6  P<0.05 | N.S. | N.S. | TgR > FVB/N |

Comparisons were made using two-way analysis of variance (ANOVA) with one factor being the transgene effect and the drug effect. Entries display ANOVA test results and post-hoc Neumann-Keuls (N.K.) comparisons. Abbreviations: N.S. = main effect or interaction not significant.
