# Supplementary figures and images for: Cholinergic Stress Signals Accompany MicroRNA-Associated Stereotypic Behavior and Glutamatergic Neuromodulation in the Prefrontal Cortex
Source: Biomolecules. 2020 Jun 3;10(6):848. doi: 10.3390/biom10060848 (PMC7355890; doi:10.3390/biom10060848)

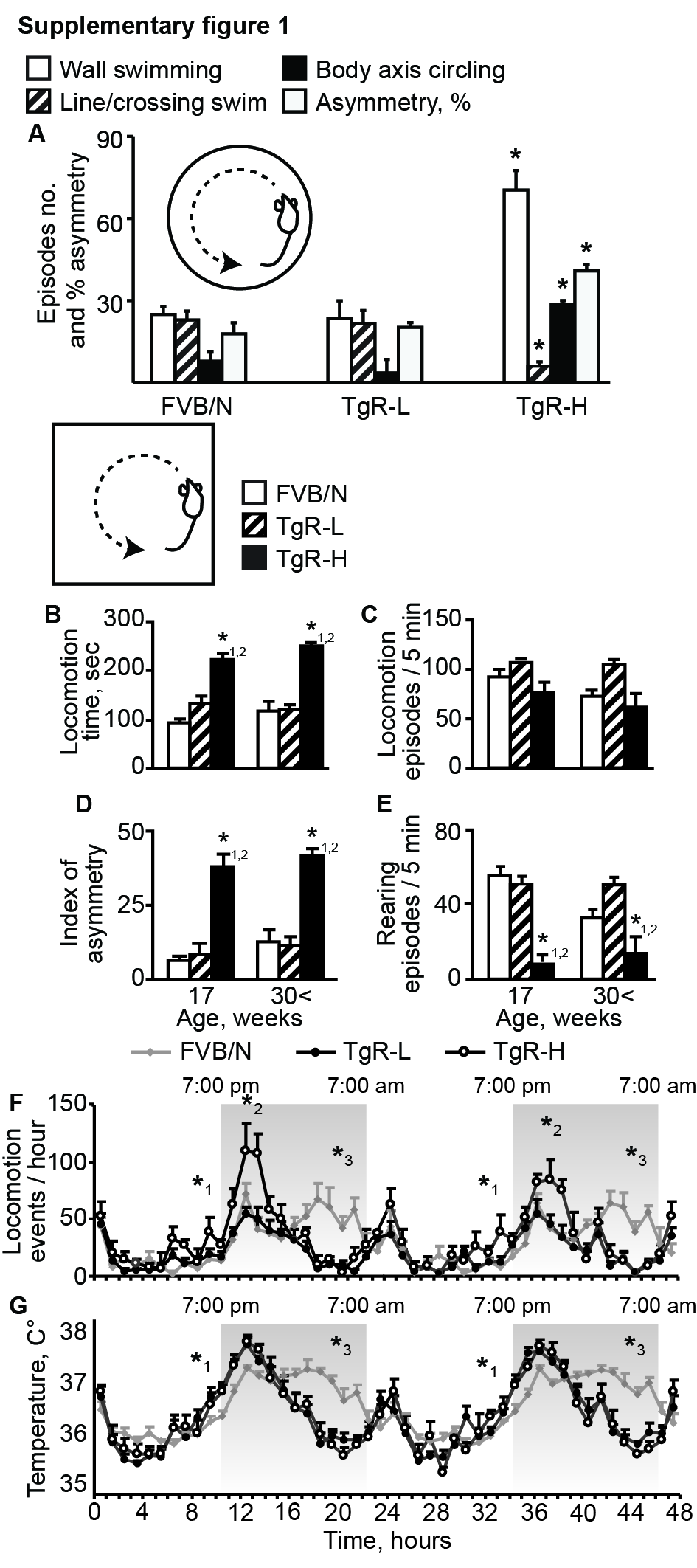

Supplement: Supplementary file 1 [file biomolecules-10-00848-s001.zip › Supplementary figure 1-01.tif]
